# Supplementary material for: Storage-Dependent Generation of Potent Anti-ZIKV Activity in Human Breast Milk
Source: Viruses. 2019 Jun 28;11(7):591. doi: 10.3390/v11070591 (PMC6669682; doi:10.3390/v11070591)
Supplement: Supplementary file 1 [file viruses-11-00591-s001.pdf]

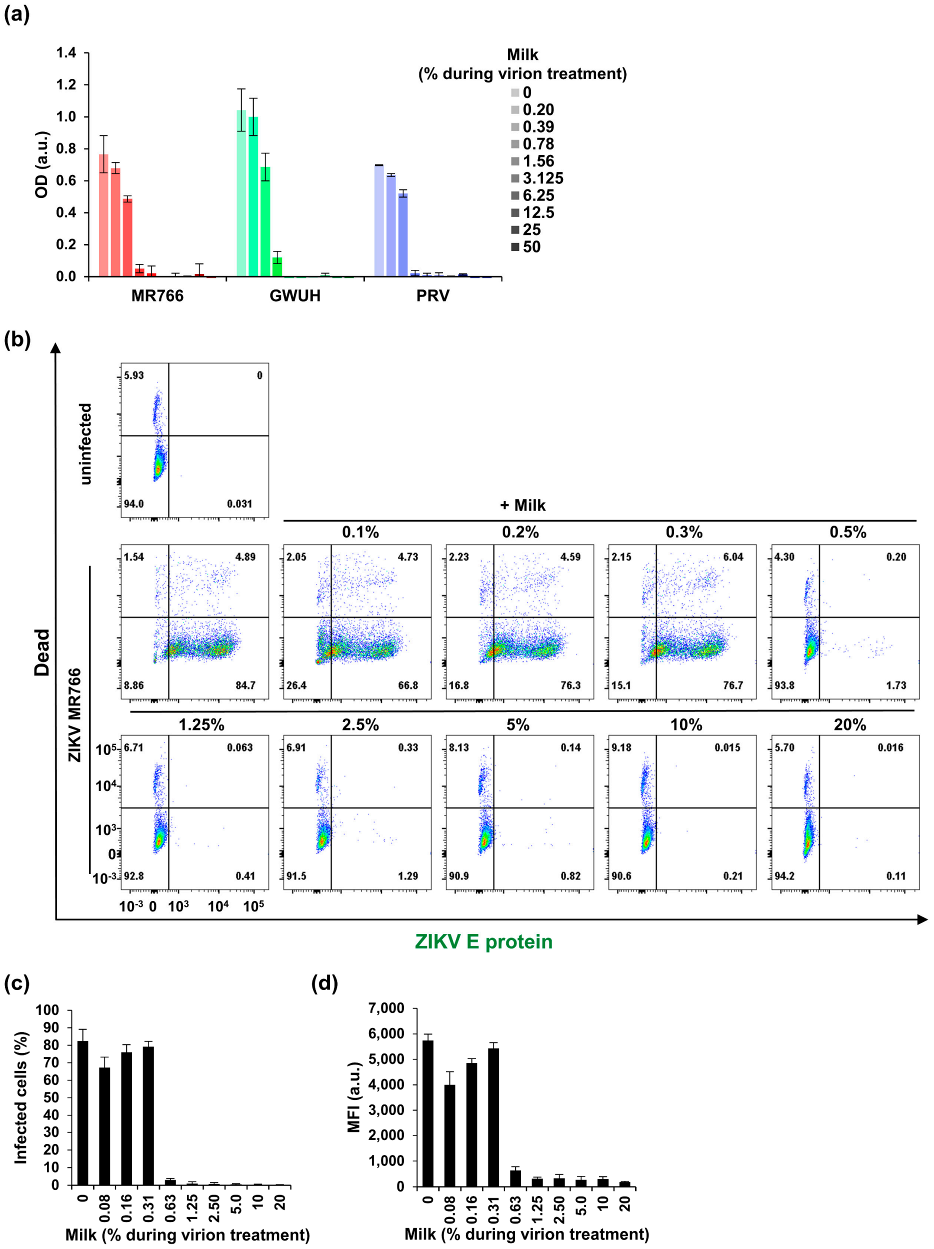

Figure S1. The effect of long-term freeze-stored breast milk on ZIKV infection. (a) ZIKV MR766, GWUH, or PRV (MR766: 1.58, GWUH: 2.51, PRV: 1.58×10^{7} TCID$_{50}$/ml) were mixed 1:1 with thawed pooled breast milk at indicated concentrations. Mixtures were then diluted 10-fold onto Vero E6 cells and incubated for 2 hours before medium was changed. Infection rates were determined 2 days later by a cell-based immunodetection assay that enzymatically quantifies the flavivirus protein E. Optical density (OD) of the converted substrate was determined at 450 nm in a microplate reader as arbitrary units (a.u.). (Find normalized data in Fig. 1a). **(b)** ZIKV MR766 (1.58×10^{6} TCID$_{50}$/ml) was incubated with indicated concentrations of thawed pooled breast milk for 10 minutes at room temperature. Mixtures were then diluted 10-fold onto Vero E6 cells and incubated for 2 hours before medium was changed. Two days later cells were trypsinized, fixed, stained by fixable viability dye (Y-axis) and for flavivirus protein E (X-Axis). Infection rates and cell viability were analyzed by flow cytometry. Data were evaluated as **(c)** average % infected cells or **(d)** mean fluorescence intensity (MFI).

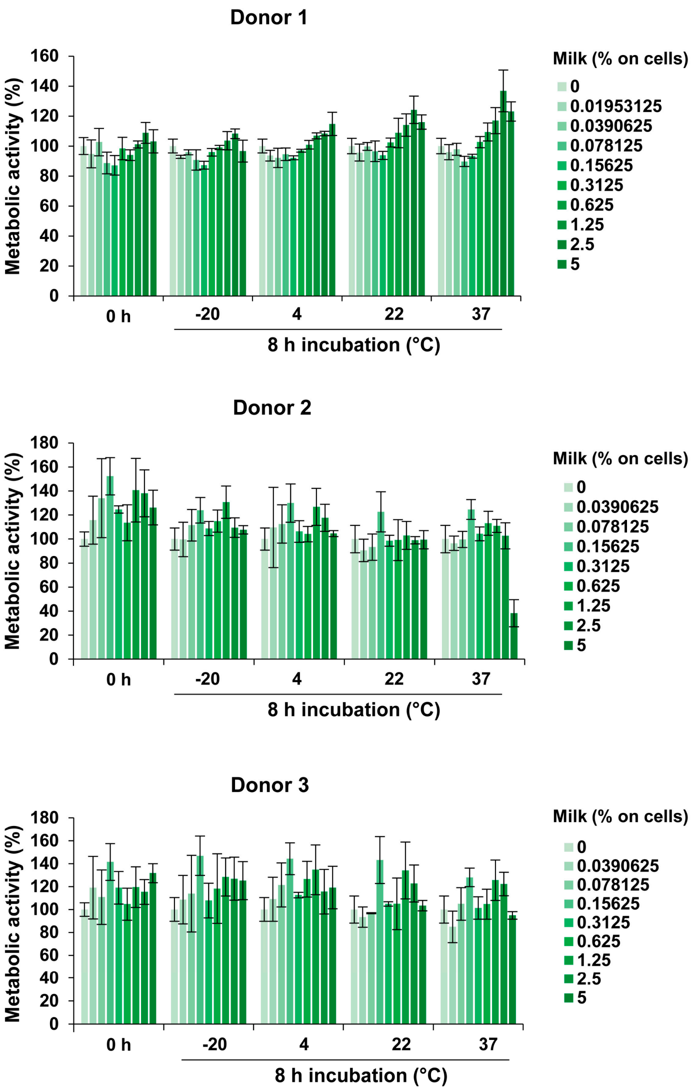

Figure S2. Cytotoxicity of the fresh and incubated breast milk samples. Breast milk was collected from 3 donors (see Fig. 5) 30 minutes after lactation and immediately used to inoculate Vero E6 cells at indicated concentrations. Additionally, milk was stored at -20°C, 4°C, 22°C, or 37°C for 8 hours before it was added to cells. After 2 hours of incubation, medium was replaced and the cellular viability determined 2 days later by CellTiter-Glo® Luminescent Cell Viability Assay. Data are normalized to viability in the absence of the respective sample and represent average values obtained from triplicate inoculations ± standard deviations.

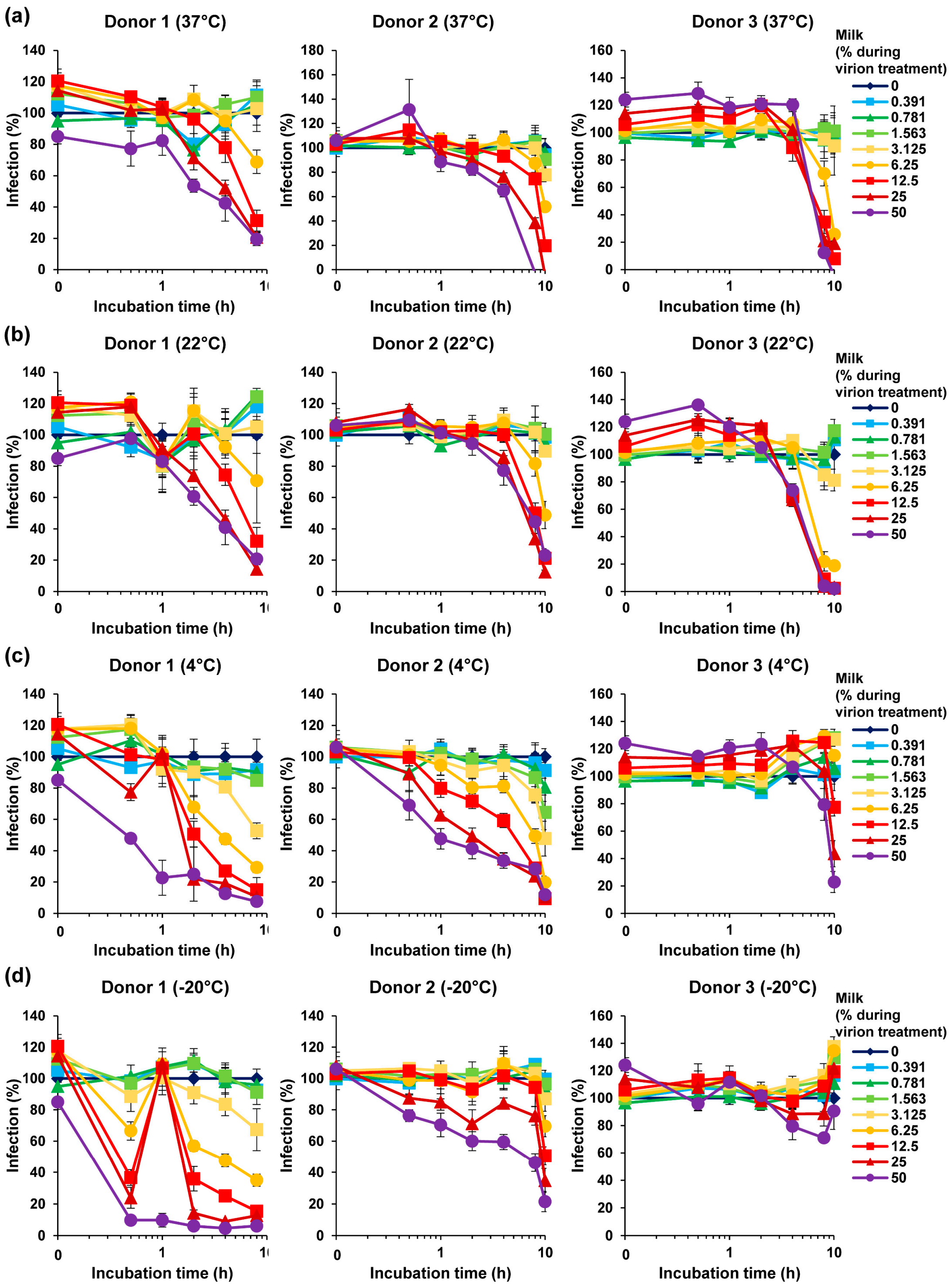

Figure S3. Kinetics of ZIKV inhibition by incubated individual breast milk samples at different temperatures. Illustrated is data from Fig. 5. Fresh breast milk was received from 3 donors and after 30 minutes mixed 1:1 with ZIKV MR766 (1.58×10^7 $TCID_{50}$/ml) at indicated concentrations and incubated for 10 minutes at room temperature. Mixtures were then diluted 10-fold onto Vero E6 cells and incubated for 2 hours before medium was changed. Additionally, the breast milk was incubated at **(a)** 37°C, **(b)** 22°C, **(c)** 4°C, or **(d)** -20°C for indicated time points before mixing with ZIKV and inoculation of Vero E6 cells. Infection rates were determined 2 days later by a cell-based immunodetection assay that enzymatically quantifies the flavivirus protein E. Infection data are normalized to infection rates in the absence of the respective breast milk sample and represent average values obtained from triplicate infections ± standard deviations.

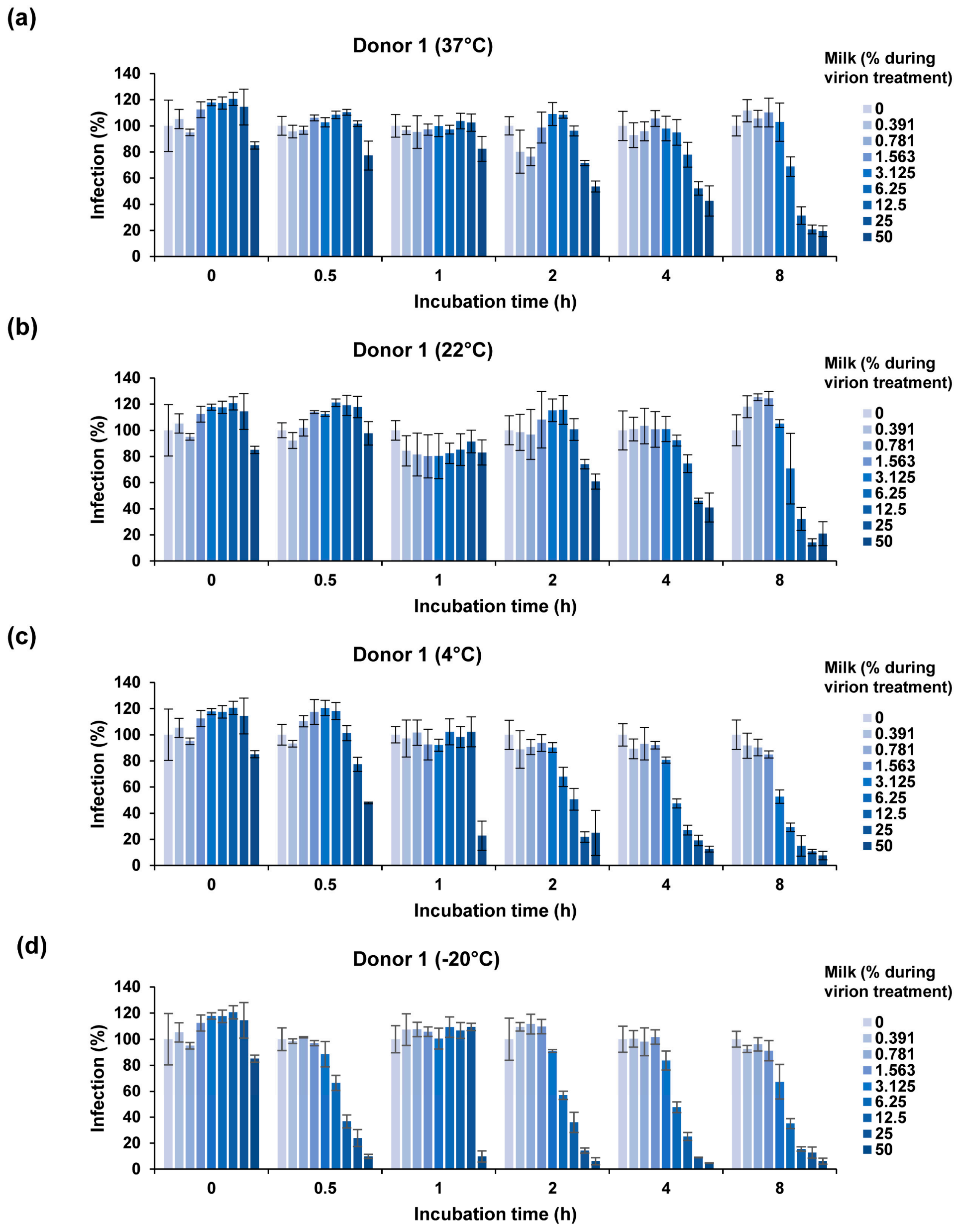

Figure S4. Bar diagram of dose-dependent ZIKV inhibition by incubated breast milk sample of donor 1. Illustrated is data from Fig. 5 left panels. 30 minutes after donation fresh breast milk was mixed 1:1 with ZIKV MR766 (1.58×10^{7} $TCID_{50}$/ml) at indicated concentrations and incubated for 10 minutes at room temperature. Mixtures were then diluted 10-fold onto Vero E6 cells and incubated for 2 hours before medium was changed. Additionally, the breast milk was incubated at **(a)** 37°C, **(b)** 22°C, **(c)** 4°C, or **(d)** -20°C for indicated time points before mixing with ZIKV and inoculation of Vero E6 cells. Infection rates were determined 2 days later by a cell-based immunodetection assay that enzymatically quantifies the flavivirus protein E. Infection data are normalized to infection rates in the absence of the respective breast milk sample and represent average values obtained from triplicate infections ± standard deviations.

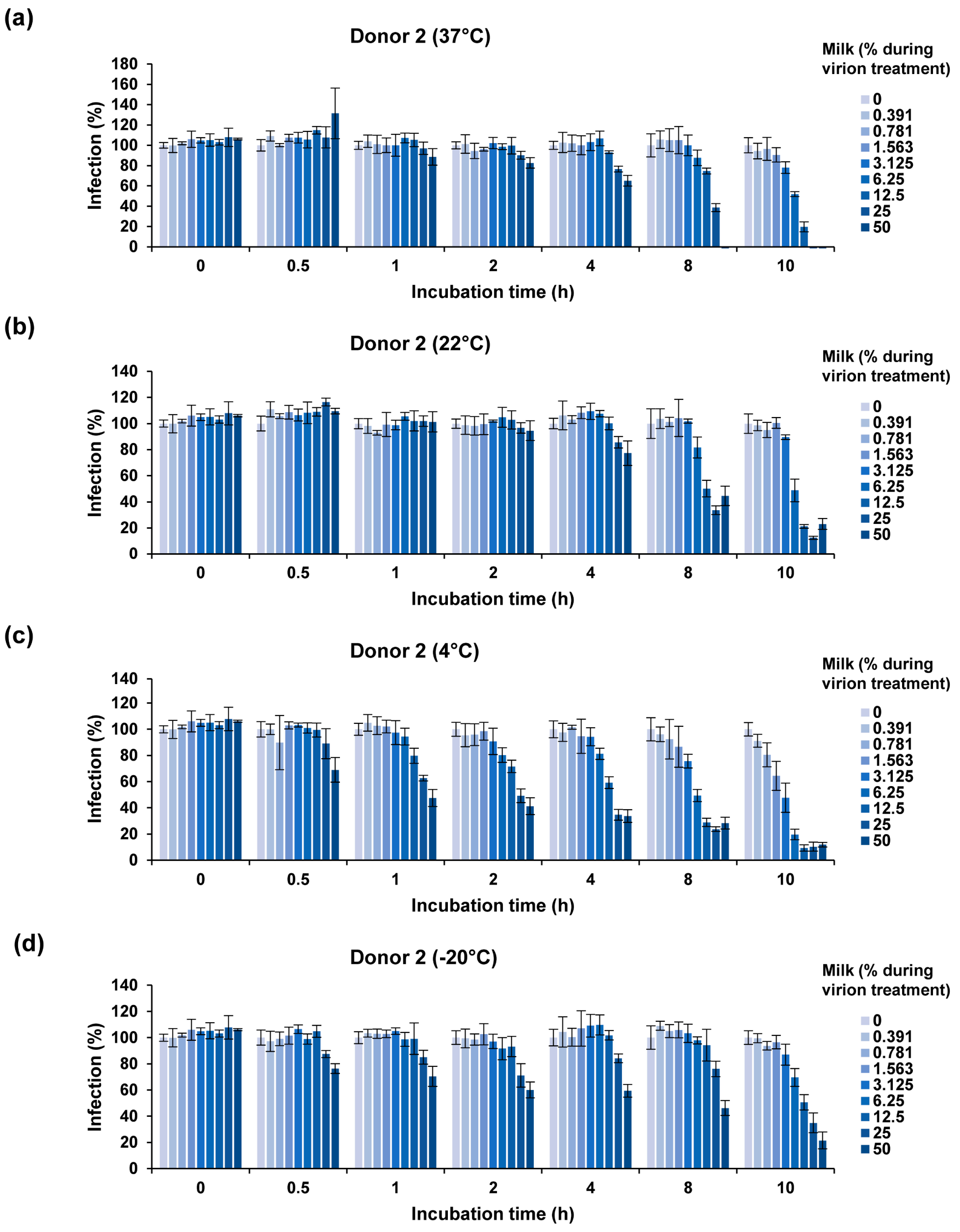

Figure S5. Bar diagram of dose-dependent ZIKV inhibition by incubated breast milk sample of donor 2. Illustrated is data from Fig. 5 middle panels. 30 minutes after donation fresh breast milk was mixed 1:1 with ZIKV MR766 (1.58×10^7 $TCID_{50}$/ml) at indicated concentrations and incubated for 10 minutes at room temperature. Mixtures were then diluted 10-fold onto Vero E6 cells and incubated for 2 hours before medium was changed. Additionally, the breast milk was incubated at **(a)** 37°C, **(b)** 22°C, **(c)** 4°C, or **(d)** -20°C for indicated time points before mixing with ZIKV and inoculation of Vero E6 cells. Infection rates were determined 2 days later by a cell-based immunodetection assay that enzymatically quantifies the flavivirus protein E. Infection data are normalized to infection rates in the absence of the respective breast milk sample and represent average values obtained from triplicate infections ± standard deviations.

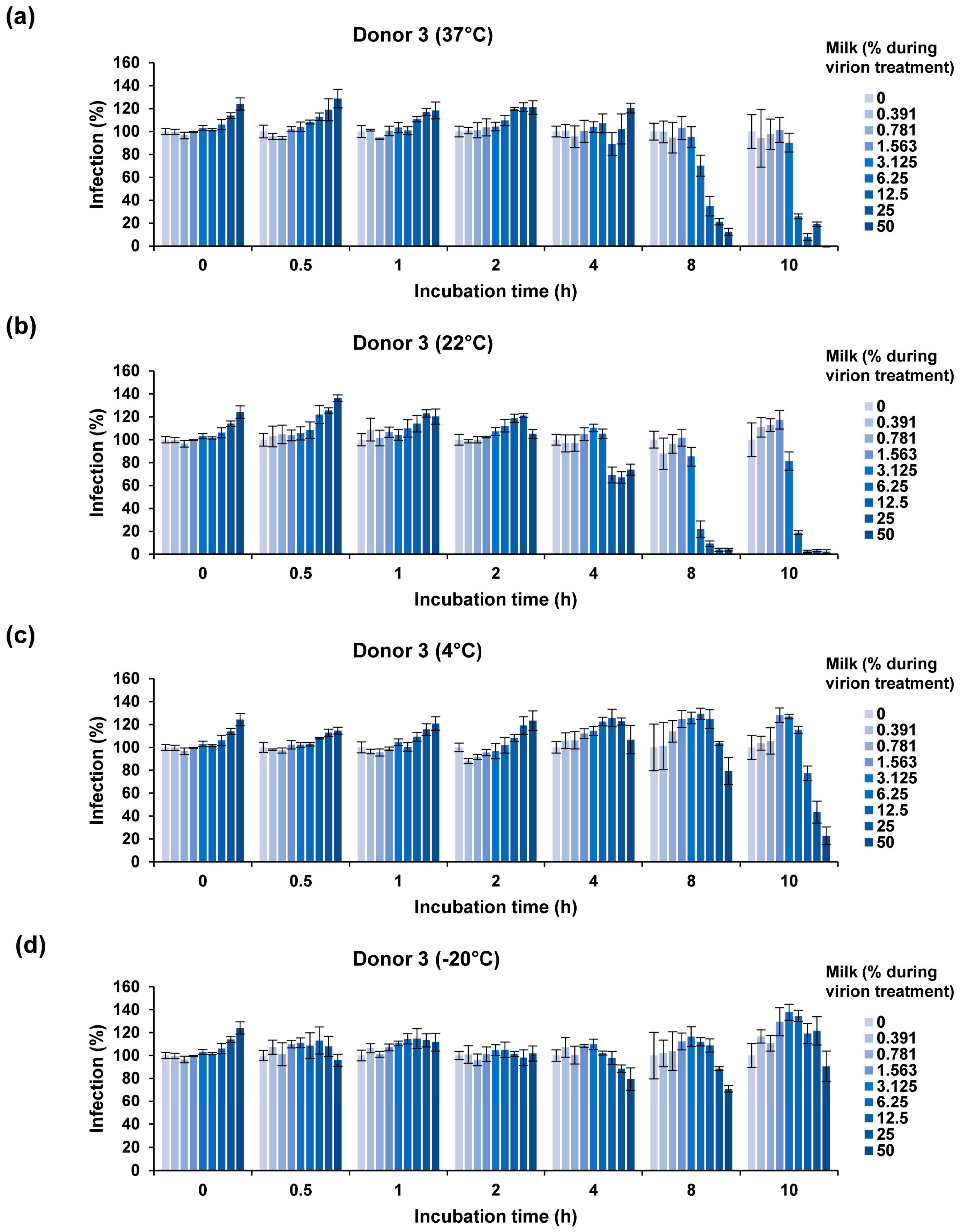

Figure S6. Bar diagram of dose-dependent ZIKV inhibition by incubated breast milk sample of donor 3. Illustrated is data from Fig. 5 right panels. 30 minutes after donation fresh breast milk was mixed 1:1 with ZIKV MR766 (1.58×10^{7} $TCID_{50}$/ml) at indicated concentrations and incubated for 10 minutes at room temperature. Mixtures were then diluted 10-fold onto Vero E6 cells and incubated for 2 hours before medium was changed. Additionally, the breast milk was incubated at **(a)** 37°C, **(b)** 22°C, **(c)** 4°C, or **(d)** -20°C for indicated time points before mixing with ZIKV and inoculation of Vero E6 cells. Infection rates were determined 2 days later by a cell-based immunodetection assay that enzymatically quantifies the flavivirus protein E. Infection data are normalized to infection rates in the absence of the respective breast milk sample and represent average values obtained from triplicate infections ± standard deviations.
